# Прогностическое значение исследования уровня С-пептида в оценке риска развития сахарного диабета 1 типа у детей

**DOI:** 10.14341/probl13645

**Published:** 2026-05-20

**Authors:** К. Г. Корнева, Д. А. Чичеватов, О. Б. Безлепкина, Л. Г. Стронгин, В. Е. Загайнов

**Affiliations:** Приволжский исследовательский медицинский университет; Privolzhsky Research Medical University; Пензенский государственный университет; Penza state University; Национальный медицинский исследовательский центр эндокринологии им. академика И.И. Дедова; Endocrinology Research Centre

**Keywords:** сахарный диабет 1 тип, сибс, С-пептид, прогнозирование, type 1 diabetes mellitus, sibling, C-peptide, prediction

## Abstract

**ОБОСНОВАНИЕ:**

ОБОСНОВАНИЕ. Сахарный диабет 1 типа (СД1) — хроническое аутоиммунное заболевание, развивающееся в результате деструкции β-клеток поджелудочной железы с формированием инсулиновой недостаточности, маркером которой является уровень С-пептида. Секреция С-пептида исследовалась в диагностическом, прогностическом и терапевтическом аспектах у пациентов с уже манифестированным СД1. Состояние инсулиновой секреции, оцениваемой по уровню С-пептида на доклинической стадии заболевания, остается наименее изученным вопросом.

**ЦЕЛЬ:**

ЦЕЛЬ. Целью настоящего исследования была оценка возможности прогнозирования развития СД1 у здоровых сибсов на основании динамики концентрации С-пептида.

**МАТЕРИАЛЫ И МЕТОДЫ:**

МАТЕРИАЛЫ И МЕТОДЫ. Проведено многоцентровое проспективное продольное исследование с участием 532 человек. Медиана наблюдения — 5,6 года. Группа 1 (n=325) включала детей в возрасте от 0 до 18 лет с впервые выявленным СД1 , группа 2 (n=201) — здоровых братьев и сестер (сибсы), не заболевших СД1 до момента окончания исследования, группа 3 (n=6) — здоровых сибсов, заболевших до завершения исследования. Всем участникам выполнено динамическое исследование анализа крови на С-пептид методом твердофазного хемилюминесцентного иммyноанализа.

**РЕЗУЛЬТАТЫ:**

РЕЗУЛЬТАТЫ. В группе 1 и 3 фактическая медиана концентрации исходного уровня С-пептида находилась ниже референсных значений: 0,31 нг/мл [95% ДИ 0,10–1,39] и 0,56 нг/мл [95% ДИ 0,32–0,85] соответственно, в группе 2 она соответствовала нижней границе референсных значений: 0,88 нг/мл [95% ДИ 0,28–2,69]. Наблюдаемые различия были статистически значимы для групп 1 и 2 (χ2 =168,29, df=1, p<0,001) и для групп 2 и 3 (χ2 =4,2292, df=1, p=0,040). При регрессионном моделировании обнаружена нелинейная положительная зависимость концентрации С-пептида от возраста. В любой возрастной категории медиана инициальной концентрации С-пептида в группах 1 и 3 была ниже, а в группе 2 выше среднего уровня (Intercept), характерного для когорты конкретного возраста. Ассоциация концентрации С-пептида и времени с момента начала наблюдения была статистически значимой (p<0,05) и разнонаправленной: со временем концентрация снижалась в группах 1 и 3 и возрастала в группе 2.

**ЗАКЛЮЧЕНИЕ:**

ЗАКЛЮЧЕНИЕ. Измерение исходного уровня С-пептида с последующим его динамическим контролем может быть дополнительным скрининговым инструментом для прогнозирования развития СД1 у здоровых сибсов.

## Abstract (en)

**BACKGROUND:**

BACKGROUND: Type 1 diabetes mellitus (T1DM) is a chronic progressive autoimmune disease characterized by destruction of pancreatic betta cells leading to insulin deficiency, measured by C-peptide testing. C-peptide secretion has been studied in diagnostic, prognostic and therapeutic aspects in patients with already manifested T1DM. Insulin secretion assessed by the level of C-peptide at the preclinical stage remains under-studied.

**AIM:**

AIM: The aim of the study was to assess the possibility of predicting the development of T1DM in healthy siblings based on dynamic measurements of C-peptide concentration.

**MATERIALS AND METHODS:**

MATERIALS AND METHODS: A multicenter prospective longitudinal study involving 532 individuals was conducted. Median follow-up duration was 5.6 years. Group 1 (n=325) included children aged 0–18 years with newly diagnosed T1DM; group 2 (n=201) consisted of healthy brothers and sisters (siblings) who did not develop T1DM until the end of the study; group 3 (n=6) comprised healthy siblings who developed T1DM before the study completion. All participants underwent dynamic testing of blood analysis for C-peptide using solid-phase chemiluminescent immunoassay.

**RESULTS:**

RESULTS: In groups 1 and 3, the actual median concentration of baseline C-peptide was below reference values: 0.31 ng/mL [95% CI 0.10–1.39] and 0.56 ng/mL [95% CI 0.32–0.85], respectively. In group 2, it corresponded to the lower limit of reference values: 0.88 ng/mL [95% CI 0.28–2.69]. The observed differences were statistically significant between groups 1 and 2 (χ2 =168.29, df=1, p < 0.001) and between groups 2 and 3 (χ2 =4.2292, df=1, p=0.040). Regression modeling revealed a nonlinear positive relationship between C-peptide concentration and age. At any age category, the median initial C-peptide concentration in groups 1 and 3 was lower than that in group 2 above the average level ("intercept") characteristic for the cohort of specific age. The association between C-peptide concentration and time since observation onset was statistically significant (p < 0.05) and bidirectional: over time, concentrations decreased in groups 1 and 3 and increased in group 2.

**CONCLUSION:**

CONCLUSION: Measurement of baseline C-peptide levels followed by dynamic monitoring may serve as an additional screening tool for predicting the risk of T1DM development in healthy siblings.

## ОБОСНОВАНИЕ

Сахарный диабет 1 типа (СД1) — распространенное хроническое заболевание, развивающееся в результате аутоиммунной деструкции β-клеток поджелудочной железы с формированием инсулиновой недостаточности, хорошо известным маркером которой служит уровень С‑пептида [1]. Традиционно С‑пептид широко используется в клинической практике для дифференциальной диагностики типов сахарного диабета [2]. Уровень C-пептида при установлении диагноза СД1 может быть полезен для определения функционального состояния инсулин-продуцирующей функции β-клеток в краткосрочном периоде [3]. Изучены долгосрочные траектории содержания С‑пептида у пациентов с СД1, характеризующиеся неуклонным его снижением в первые годы болезни с последующей фазой стабилизации [4–5]. Сохраненный уровень С‑пептида может быть предиктором продолжительности клинической ремиссии СД1 в начале заболевания [6]. С‑пептид используется для оценки эффективности иммуномодулирующей терапии, направленной на увеличение продолжительности доклинической фазы течения СД1 [7–8]. Сохраняющаяся остаточная секреция С‑пептида ассоциирована с лучшим метаболическим контролем сахарного диабета [9]. С‑пептид может оказывать собственное физиологическое действие, участвуя в замедлении развития микрососудистых осложнений, что стало поводом для его использования в качестве экспериментального терапевтического средства [10–11]. Определение уровня С‑пептида в сочетании с аутоантителами (ААТ) при впервые возникшем СД1 позволяет классифицировать заболевших на фенотипы, служащие основой для прогнозирования клинического течения болезни в будущем [12].

В большинстве опубликованных исследований секреция С‑пептида оценивалась в диагностическом, прогностическом и терапевтическом аспектах у пациентов с уже манифестировавшим СД1. Состояние инсулиновой секреции, оцениваемой по уровню С‑пептида на доклинической стадии заболевания, остается наименее изученным вопросом.

## ЦЕЛЬ ИССЛЕДОВАНИЯ

Целью настоящего исследования была оценка возможности прогнозирования развития СД1 у здоровых сибсов на основании динамики концентрации С‑пептида.

## МАТЕРИАЛЫ И МЕТОДЫ

## Место и время проведения исследования

Место проведения. Исследование проведено в эндокринологических отделениях ГБУ здравоохранения Нижегородской области «Нижегородская областная детская клиническая больница», Бюджетное учреждение Чувашской республики «Республиканская детская клиническая больница»Министерства здравоохранения Чувашской республики, ГБУ Республики Марий Эл «Детская республиканская клиническая больница».

Время исследования. Набор пациентов в исследование проходил с октября 2017 г. по ноябрь 2020 г. Срок окончания периода наблюдения — февраль 2025 г.

## Изучаемые популяции (одна или несколько)

Изучено две популяции пациентов: пациенты с впервые выявленным СД1 и их здоровые братья и сестры (сибсы).

Критерии включения: Критерии включения для популяции пациентов с СД1: возраст 0–17 лет, диагноз СД1 установлен согласно диагностическим критериям (ВОЗ, 1999) и наличию положительных ААТ. Для популяции здоровых сибсов: братья и сестры пациентов с СД1 (потомки одних родителей), уровень глюкозы крови и HbA1c ниже диагностического порога СД1.

Критерии исключения: Критерии исключения для популяции пациентов с СД1: наличие других типов сахарного диабета.

## Способ формирования выборки из изучаемой популяции (или нескольких выборок из нескольких изучаемых популяций)

Выборка формировалась сплошным методом.

## Дизайн исследования

Многоцентровое наблюдательное динамическое проспективное двухвыборочное неконтролируемое сравнительное.

## Описание медицинского вмешательства (для интервенционных исследований)

Не было.

## Методы

Лабораторная диагностика предполагала определение уровня HbA1c и С‑пептида. У пациентов с впервые выявленным СД1 тестирование уровня С‑пептида проводилось при включении в исследование и каждые три месяца в течение 1 года, у здоровых сибсов — ежегодно в течение трех лет. Забор материала проводился в локальных учреждениях. Доставка образцов крови осуществлялась с соблюдением необходимых условий транспортировки в независимую централизованную лабораторию, сертифицированную согласно Европейским стандартам. HbA1c оценивали методом высокоэффективной жидкостной хроматографии на анализаторе VARIANT IV TURBO (Bio-RAD, США/Франция). Референсные значения 4–6,2%. С‑пептида — методом твердофазного хемилюминесцентного иммyноанализа на анализаторе IMMULITE 2000XPi (Siemens Healthcare Diagnostics, США). Референсные значения 0,9–7,1 нг/мл.

## Статистический анализ

Все статистические вычисления проводились с использованием свободно распространяемого языка программирования R (v. 4.4.1) и интегрированной среды разработчика RStudio 2024.09.1 Build 394 © 2009-2024 Posit Software, PBC. Описательные статистики оценивались с помощью базовых встроенных библиотек RStudio. В качестве центральных характеристик применялись среднее, стандартное отклонение и медиана с 95% ДИ. В исследовании применялась обобщенная линейная регрессия (Generalized Linear Regression Models, GLM). Изменяющаяся во времени концентрация С‑пептида была зависимой переменной. В качестве предикторов рассматривались: 1) группа, в которую был включен участник; 2) время измерения концентрации С‑пептида; 3) пол; 4) возраст. Был применен математический анализ Байеса, реализованный в пакете brms (Bayesian Regression Models using ‘Stan’, v. 2.21.0, https://github.com/paul-buerkner/brms). Поскольку концентрация С‑пептида принимала только неотрицательные значения, гамма-распределение рассматривалось в качестве функции плотности распределения отвечающей переменной. Некоторые регрессионные модели подразумевали индивидуальную вариабельность (random-effects models), в связи с чем они были многоуровневыми с индивидуальной константой (multilevel model with individual intercept). Байесовский подход предполагал только интервальную (доверительный интервал, ДИ), оценку вариации параметров моделей. Для оценки использовался 95% ДИ. Критерий Краскела-Уоллиса χ² применялся для сравнения более двух независимых выборок. Дополнительно мы использовали bayestestR (https://easystats.github.io/bayestestR/) и ggplot2 (https://ggplot2.tidyverse.org, https://github.com/tidyverse/ggplot2) свободно распространяемые программные пакеты. Все графические иллюстрации выполнены на основе последнего. Первичные данные включали пропущенные значения. Статистическое восстановление данных не проводилось. Все вычисления осуществлялись только с полными данными.

## Этическая экспертиза

Протокол исследования одобрен локальным комитетом по этике ГБУЗ НО «Нижегородская областная детская клиническая больница» (выписка из протокола №30 от 06.10.2017). Родители пациентов подписывали информированное согласие до включения в исследование.

## РЕЗУЛЬТАТЫ

В исследование были включены 532 участника, из них 325 имели впервые выявленный СД1 и 207 были здоровые сибсы. Возраст варьировал от 3 месяцев до 28 лет, средний возраст составил 9,1±5,4 года. У 95% наблюдаемых возраст не превышал 18 лет, 25 человек были старше 18 лет, так как были включены в качестве старших здоровых сибсов. Среди участников доля лиц женского пола составила 41,7% (222), мужского — 58,3% (310). Из Нижегородской области в исследовании прияли участие 412 человек (77,4%), из Чувашской Республики — 95 (17,9%), из Республики Марий Эл — 25 (4,7%). Шесть человек из группы здоровых сибсов заболели в процессе наблюдения.

Все пациенты были разделены на 3 группы: группа 1 (n=325) — дети, с впервые выявленным СД1; группа 2 (n=201) — здоровые сибсы, одновременно включенные в исследование и не заболевшие СД1 до момента окончания исследования; группа 3 (n=6) — сибсы, одновременно включенные в исследование, и заболевшие СД1 до момента окончания исследования.

## Исходные показатели уровня С‑пептида при включении в исследование

Для групп 1 и 2 отличие было значимым (χ²=168,29, df=1, p<0,001), для групп 2 и 3 отличие также было значимым (χ²=4,2292, df=1, p=0,040), для групп 1 и 3 значимых отличий не обнаружено (χ²=3,7654, df=1, p=0,052) (рис. 1).

**Figure:**
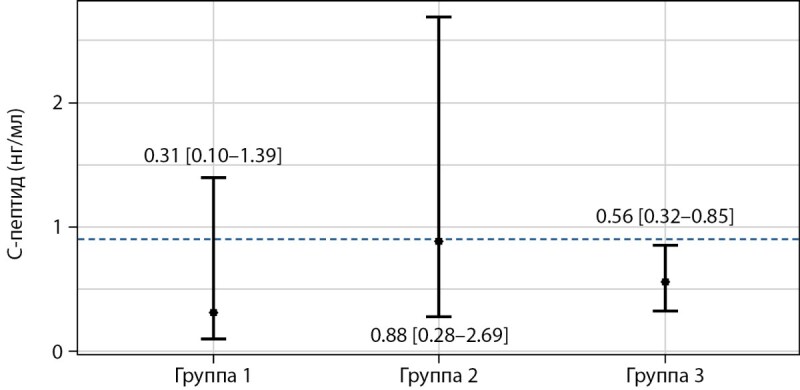
Рисунок 1. Распределение исходных значений уровня С‑пептида у пациентов разных групп.Пунктирная линия — нижняя граница референсного интервала соответствует 0,9 нг/мл.

Ассоциации исходной концентрации С‑пептида с полом не выявлено (χ²=0,10009, df=1, p=0,7517). Зависимость исходной концентрации С‑пептида от возраста в группе 2 представлена на рисунке 2.

**Figure:**
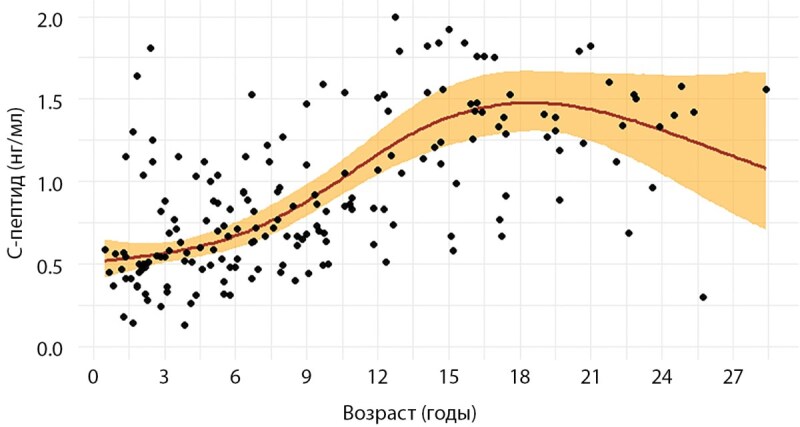
Рисунок 2. Модель зависимости концентрации С‑пептида от возраста в группе 2.Черными точками показаны фактические значения исходного уровня С‑пептида.

Прослеживается нелинейное возрастание концентрации до возраста 18 лет. Используя уравнение этой кривой, были рассчитаны ожидаемые нормальные уровни С‑пептида для каждого возраста от 1 до 18 лет у здоровых сибсов (таблица 1). Поскольку дети остались здоровы до конца наблюдения, эти значения могли рассматриваться как референсные.

**Table:** Таблица 1. Ожидаемые медианы концентрации С‑пептида в группе 2

Возраст, лет	С‑пептид, нг/мл	Q2.5	Q97.5	Возраст, лет	С‑пептид, нг/мл	Q2.6	Q97.6
1	0,64	0,52	0,78	10	1,07	0,96	1,18
2	0,65	0,56	0,75	11	1,17	1,06	1,29
3	0,66	0,59	0,74	12	1,28	1,15	1,41
4	0,68	0,62	0,75	13	1,37	1,22	1,53
5	0,71	0,64	0,79	14	1,45	1,29	1,63
6	0,76	0,67	0,85	15	1,52	1,35	1,72
7	0,82	0,73	0,92	16	1,57	1,39	1,77
8	0,89	0,79	0,99	17	1,60	1,42	1,81
9	0,97	0,87	1,08	18	1,62	1,44	1,83

Результат моделирования исходного уровня С‑пептида в зависимости от принадлежности пациента к одной из трех исследованных групп с учетом возраста ребенка представлен на рисунке 3 и в таблице 2. Была построена регрессионная модель, в которой исходный уровень С‑пептида был зависимой переменной, а исследуемая группа и возраст выступали в качестве предикторов.

**Figure:**
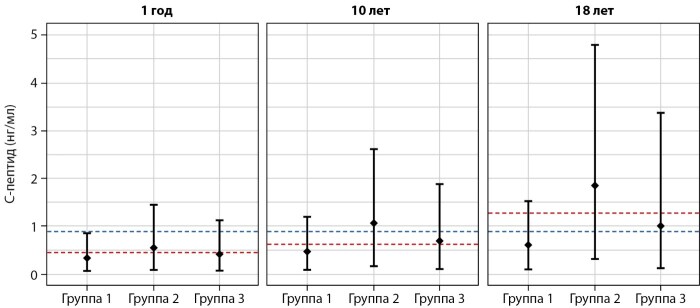
Рисунок 3. Модель зависимости исходной концентрации С‑пептида от исследуемой группы (для трех возрастов).Голубая пунктирная линия — нижняя граница референсного интервала С‑пептида соответствует 0,9 нг/мл. Красная пунктирная линия — константа регрессионной модели (Intercept), усредненное значение для каждого возраста (0,47, 0,64, 1,21 нг/мл соответственно).

**Table:** Таблица 2. Прогнозируемые исходные медианы концентрации С‑пептида в зависимости от группы и возраста ребенка

Возраст, лет	С‑пептид, нг/мл	Q2.5	Q97.5	Группа	Возраст, лет	С‑пептид, нг/мл	Q2.6	Q97.6	Группа
1	0,33	0,06	0,84	Группа 1	10	0,47	0,09	1,16	Группа 1
0,55	0,10	1,37	Группа 2	1,03	0,19	2,61	Группа 2
0,42	0,08	1,10	Группа 3	0,67	0,11	1,82	Группа 3
2	0,34	0,06	0,87	Группа 1	11	0,50	0,09	1,25	Группа 1
0,57	0,10	1,45	Группа 2	1,16	0,22	2,98	Группа 2
0,42	0,07	1,10	Группа 3	0,74	0,12	2,18	Группа 3
3	0,35	0,06	0,92	Группа 1	12	0,52	0,09	1,29	Группа 1
0,58	0,10	1,46	Группа 2	1,33	0,23	3,37	Группа 2
0,44	0,07	1,18	Группа 3	0,81	0,13	2,38	Группа 3
4	0,36	0,07	0,89	Группа 1	13	0,54	0,10	1,32	Группа 1
0,61	0,11	1,56	Группа 2	1,46	0,29	3,66	Группа 2
0,46	0,08	1,22	Группа 3	0,87	0,13	2,62	Группа 3
5	0,37	0,07	0,94	Группа 1	14	0,57	0,11	1,41	Группа 1
0,63	0,13	1,57	Группа 2	1,60	0,29	4,10	Группа 2
0,46	0,08	1,20	Группа 3	0,91	0,14	2,71	Группа 3
6	0,38	0,07	0,98	Группа 1	15	0,58	0,10	1,52	Группа 1
0,69	0,11	1,75	Группа 2	1,72	0,30	4,17	Группа 2
0,50	0,08	1,34	Группа 3	0,96	0,14	3,07	Группа 3
7	0,40	0,07	0,99	Группа 1	16	0,58	0,10	1,45	Группа 1
0,74	0,13	1,85	Группа 2	1,80	0,29	4,53	Группа 2
0,52	0,09	1,39	Группа 3	0,97	0,15	3,03	Группа 3
8	0,42	0,08	1,03	Группа 1	17	0,58	0,10	1,46	Группа 1
0,81	0,14	2,03	Группа 2	1,88	0,30	4,74	Группа 2
0,56	0,09	1,59	Группа 3	1,00	0,14	3,24	Группа 3
9	0,44	0,08	1,10	Группа 1	18	0,58	0,10	1,48	Группа 1
0,92	0,15	2,34	Группа 2	1,83	0,32	4,87	Группа 2
0,61	0,10	1,67	Группа 3	1,01	0,14	3,11	Группа 3

Модель разграничивала исходные значения уровня С‑пептида в группах, при этом с возрастом это различие увеличивалось. Группа 3 занимала промежуточное положение. Медиана концентрации исходного уровня показателя в этой группе колебалась в области среднего исходного уровня во всей исследуемой когорте (Intercept). В группе 1 медиана концентрации находилась ниже средней величины (Intercept) во всей исследованной когорте, в группе 2 — выше. В группе 2 медиана концентрации достигла превышения нижнего референсного значения начиная с 9 лет, а в группе 3 — с 14 лет. В группе 1 медиана концентрации С‑пептида всегда была ниже референсного интервала.

## Динамика концентрации С‑пептида

Для изучения динамики концентрации С‑пептида использовались три регрессионные модели, построенные отдельно для каждой группы. Предполагалось, что фиксированный момент времени повторных измерений, используемый в качестве предиктора, будет иметь разнонаправленный эффект в группах. Поскольку возраст имел значение, он рассматривался в качестве второго предиктора. Характер изменений концентрации С‑пептида относительно времени повторных измерений представлен на рисунке 4.

**Figure:**
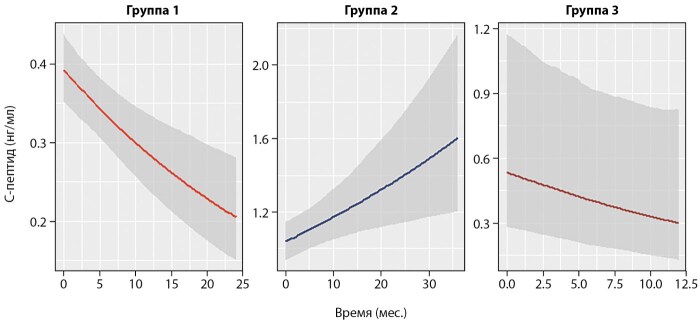
Рисунок 4. Графики независимых (маргинальных) эффектов времени.

В группах 1 и 3 наблюдалась отрицательная взаимосвязь между предикторами. С течением времени концентрация С‑пептида снижалась, причем данная ассоциация являлась статистически значимой. В группе 2 регистрировалось постепенное увеличение концентрации С‑пептида. Подобная динамика также была статистически значима.

## ОБСУЖДЕНИЕ

Исследование уровня С‑пептида важно с точки зрения прогностического значения данного лабораторного теста. Ключевым моментом является дискриминация пациентов с высоким риском развития СД1 (группа 3) и пациентов с условно низким риском (группа 2), что в перспективе позволит применять терапевтические вмешательства с целью профилактики развития заболевания. Группа 1, тем не менее, была включена в исследование, поскольку абсолютные значения и динамика концентрации С‑пептида в ней могут выступать в качестве базового ориентира для оценки степени риска заболевания у здоровых сибсов. В группе заболевших детей регистрировалась достоверно более низкая исходная концентрация С‑пептида по сравнению со здоровыми сибсами, что может быть ассоциировано с более быстрым прогрессированием СД1 [13].

Настоящая работа показала, что абсолютные значения уровня С‑пептида, измеряемые в начале наблюдения за клинически здоровым ребенком, имеют ограниченное прогностическое значение. Это связано с тем, что, во-первых, концентрация С‑пептида имеет тенденцию увеличиваться с возрастом, поэтому сложно говорить об общих референсных значениях. Во-вторых, она имеет большую вариацию. Доверительные интервалы всех исследуемых групп пересекаются, что делает невозможным идентифицировать точные дискриминационные уровни концентрации. Несмотря на то, что и первичные данные, и моделирование концентрации С‑пептида демонстрируют значимое отличие исходных уровней в группах, это имеет недостаточную прикладную ценность. Рисунок 3 убедительно показывает, что на основании только одного измерения концентрации С‑пептида невозможно отнести исследуемого в группу высокого риска, поскольку аналогичные значения могут встречаться и в группе низкого риска.

Некоторым ориентиром могут служить медианы концентрации С‑пептида, рассчитанные для разных возрастов в группе здоровых сибсов (табл. 2). Поскольку это наивысшие центральные характеристики концентрации в исследуемой когорте, то отклонение от них в сторону снижения позволяют предположить высокий риск. Во всяком случае, таблица 2 определенно демонстрирует тот факт, что в любом возрасте потенциальные значения концентрации в группе высокого риска ниже аналогичных в группе здоровых. Концентрации С‑пептида увеличиваются с возрастом, а стандартные лабораторные референсные значения не учитывают этот фактор. Это важно понимать для интерпретации результатов, поскольку отсутствие учета возрастного фактора может привести к ошибочной оценке степени секреции инсулина в младших возрастных группах.

Оценка динамики концентрации С‑пептида существенно расширяет прогностический потенциал модели, так как здесь прослеживается достаточно устойчивая тенденция. Если у клинически здорового ребенка в процессе повторных измерений нет увеличения уровня С‑пептида, то он с высокой долей вероятности находится в группе высокого риска по сравнению с детьми, у которых наблюдалось его увеличение. Во всяком случае, индивидуальное падение уровня С‑пептида у клинически здорового ребенка может рассматриваться как крайне негативный симптом. Полученные результаты согласуются с концепцией спорадической гибели β-клеток в течение нескольких лет, предшествующих заболеванию [14].

В целом можно заключить, что комбинация абсолютного значения базового уровня С‑пептида и его динамики позволяют высказываться в пользу степени индивидуального риска развития СД1. В проспективном плацебо контролируемом исследовании профилактического влияния теплизумаба на развитие СД1 показано, что оценка уровней базового С‑пептида и глюкозы оказалась эффективной в отношении прогнозирования развития СД1 в группе высокого риска [15]. Анализ шести рандомизированных плацебо контролируемых исследований подтвердил прогрессирующее падение уровня С‑пептида в течение 24 месяцев в группе контроля у детей на ранней стадии СД1 [16].

Мониторинг концентрации C-пептида на доклинической стадии заболевания может быть полезен в прогнозировании болезни, и иметь долгосрочные преимущества, способствуя улучшению клинического течения СД1 после его дебюта, снижению частоты эпизодов диабетического кетоацидоза, сохранению повышенной эндогенной секреции инсулина, повышению информированности родителей пациентов относительно болезни [17].

Интерес к С‑пептиду сохраняется главным образом в области научных исследований, уступая его применению в рутинной практике. Накопление доказательных знаний его прогностической ценности в отношении развития СД1 в сочетании с клиническими, генетическими и иммунологическими маркерами позволит найти ему более широкое практическое применение.

## Ограничения исследования

Настоящее исследование имеет ряд ограничений, способных повлечь системные ошибки. Во-первых, в исследование не включались абсолютно здоровые дети, здоровые сибсы должны рассматриваться как экспонированная группа, поскольку они разделяют с больными родственниками целый ряд биологических свойств, включая генетическую предрасположенность. Таким образом, целевые возрастные значения концентрации С‑пептида у здоровых детей, представленные в таблице 2, могут отличаться от истинных популяционных значений.

Во-вторых, размер группы 3 (n=6) достаточно мал для проведения точных вычислений. Несмотря на применение методов Байеса, более устойчивых к феномену малых выборок, вариация в этой группе чрезвычайно велика, коэффициенты регрессии для этой группы в ряде моделей статистически незначимы. Это ограничивает правильную интерпретацию параметров, характеризующих группу. Увеличение численности данной группы позволило бы получить более точные результаты, но это требует достаточно длительного временного наблюдения.

## Направления дальнейших исследований

С учетом сложности калькуляции регрессионной модели имеет смысл сделать много комплексный анализ с использованием искусственного интеллекта, включающий повторные изменения С‑пептида в сочетании с ААТ, генетическое исследование, клинический факторы.

## ЗАКЛЮЧЕНИЕ

Измерение исходного уровня С‑пептида с последующим его динамическим контролем может быть дополнительным скрининговым инструментом для прогнозирования развития СД1 у здоровых сибсов. Настоящая работа не позволяет сделать строгих выводов. Необходимо дальнейшее исследование с расширением численности групп и применением более сложных методов компьютерного анализа с целью повышения точности прогнозирования высокого риска СД1.

## ДОПОЛНИТЕЛЬНАЯ ИНФОРМАЦИЯ

Источники финансирования. Работа выполнена по инициативе авторов без привлечения финансирования.

Конфликт интересов. Авторы декларируют отсутствие явных и потенциальных конфликтов интересов, связанных с содержанием настоящей статьи

Участие авторов. Все авторы одобрили финальную версию статьи перед публикацией, выразили согласие нести ответственность за все аспекты работы, подразумевающую надлежащее изучение и решение вопросов, связанных с точностью или добросовестностью любой части работы.
